# Cirrhotic Cardiomyopathy and Related Cardiocirculatory Dysfunction: A Systematic Review of Therapeutic Responses and Cardiorenal Outcomes

**DOI:** 10.7759/cureus.117398

**Published:** 2026-10-03

**Authors:** Sally M Nashed, Eemaz Nathaniel, Muhammad S Khan, Christian Ward-Bradley, Alaa Al-Ramahi, Fnu Sabeen, Aeyza Nasir, Rafia Saeed, Ali H Khan

**Affiliations:** 1 Forensic Medicine, Ain Shams University, Cairo, EGY; 2 Internal Medicine, University Hospital Wishaw, Wishaw, GBR; 3 Internal Medicine, Foundation University Medical College, Islamabad, PAK; 4 Ophthalmology, Belfast Health and Social Care Trust, Belfast, GBR; 5 Emergency, Ministry of Health, Makkah, SAU; 6 Internal Medicine, King Edward Medical University, Lahore, PAK; 7 Internal Medicine, Nishtar Medical College and Hospital, Multan, PAK

**Keywords:** beta-blockers, cardiac reserve, cardiocirculatory dysfunction, cirrhotic cardiomyopathy, liver cirrhosis, refractory ascites

## Abstract

Cardiocirculatory dysfunction in cirrhosis encompasses impaired cardiac reserve, diastolic dysfunction, systemic vasodilation, neurohormonal activation, and progressive reliance on compensatory cardiac activity. This systematic review evaluated how pharmacological interventions affecting cardiac function, vascular tone, effective circulating volume, or natriuretic signaling influence cardiovascular, renal, and clinical outcomes in adults with cirrhosis. MEDLINE via PubMed, Scopus, Web of Science Core Collection, and the Cochrane Central Register of Controlled Trials (CENTRAL) were searched for clinical studies published from August 1, 2016, through July 31, 2026. Eligibility was structured according to the Population, Intervention, Comparator, and Outcomes (PICO) framework, and study selection followed the Preferred Reporting Items for Systematic Reviews and Meta-Analyses 2020 (PRISMA 2020) guidelines. Five studies involving 465 participants were included in a narrative synthesis. Metoprolol did not restore impaired cardiac reserve or improve cardiac morphology in established cirrhotic cardiomyopathy. In refractory ascites, propranolol suppressed sympathetic support of systolic function, reduced renal perfusion pressure, and impaired renal function. Conversely, carvedilol-based targeted heart-rate reduction, with ivabradine added when required, improved left ventricular diastolic function and was associated with fewer episodes of acute kidney injury and hepatic encephalopathy, although mortality was not significantly reduced in the randomized comparison. Ularitide caused clinically important reductions in blood pressure, decreased urine output, and increased adverse reactions, while targeted albumin therapy did not improve cardiovascular biomarkers, renal dysfunction, or survival. The four randomized study results raised some concerns about bias, while the nonrandomized study had moderate risk of bias. These findings indicate that cardiovascular interventions in cirrhosis cannot be characterized as uniformly beneficial or harmful. Their effects may depend on disease severity, cardiac phenotype, and the extent to which sympathetic and hemodynamic compensation is required to preserve systemic and renal perfusion. Prospective, phenotype-stratified trials integrating cardiac, systemic, and renal endpoints are needed before individualized treatment strategies can be recommended.

## Introduction and background

Cirrhosis produces a complex disturbance of cardiovascular homeostasis that extends beyond portal hypertension. Progressive splanchnic vasodilation reduces effective arterial blood volume and activates the sympathetic nervous system, renin-angiotensin-aldosterone system, and other sodium-retaining pathways [1]. The resulting hyperdynamic circulation is commonly characterized by reduced systemic vascular resistance, increased heart rate, and elevated cardiac output. Although these changes initially help maintain arterial pressure and organ perfusion, sustained neurohormonal activation, altered loading conditions, systemic inflammation, and metabolic dysfunction may progressively impair myocardial performance. Cardiovascular activation in cirrhosis may therefore represent both a compensatory response and a marker of diminishing circulatory reserve [2].

Cirrhotic cardiomyopathy describes systolic or diastolic dysfunction in patients with cirrhosis without an alternative primary cardiac cause. Its recognition remains challenging because reduced afterload can preserve or exaggerate resting left ventricular ejection fraction despite limited contractile reserve [3]. The revised Cirrhotic Cardiomyopathy Consortium criteria incorporated global longitudinal strain and contemporary measures of diastolic function to improve detection of subclinical disease. Nevertheless, clinically important abnormalities may also occur outside a formal cirrhotic cardiomyopathy diagnosis, including impaired stress-induced cardiac reserve, left ventricular diastolic dysfunction, chronotropic abnormalities, sympathetic-dependent systolic activation, and circulatory underfilling or cardiac filling stress with cardiorenal consequences [4,5].

For this review, "cardiocirculatory dysfunction" was operationally restricted to these objectively assessed cardiac or circulatory abnormalities when evaluated in relation to a pharmacological intervention and a relevant cardiac, systemic-hemodynamic, renal-perfusion, neurohormonal, or clinical outcome. General descriptions of portal hypertension or hyperdynamic circulation without an intervention-linked cardiac, circulatory, or cardiorenal outcome were not sufficient for inclusion. This definition was intended to capture clinically relevant manifestations extending beyond formally diagnosed cirrhotic cardiomyopathy while limiting the inclusion of nonspecific hemodynamic alterations associated with cirrhosis.

Therapeutic management is complicated by the possibility that the same intervention may have different effects across disease stages and cardiovascular presentations. Beta-blockers reduce portal pressure but may also suppress cardiac output when sympathetic activation is required to preserve renal perfusion. Conversely, controlled heart-rate reduction may improve diastolic filling in selected patients [6]. Albumin expansion may increase circulating volume without correcting the coexistence of arterial underfilling and cardiac filling stress, while natriuretic or vasodilatory therapy may become ineffective when arterial pressure and renal autoregulation are already compromised. Existing clinical studies have evaluated these interventions using heterogeneous populations, physiological measurements, and clinical endpoints, leaving uncertainty regarding how changes in cardiac function relate to renal dysfunction, hepatic decompensation, and survival [7].

This systematic review aimed to evaluate clinical studies of pharmacological interventions that modify cardiac activity, vascular tone, effective circulating volume, or natriuretic signaling in adults with cirrhosis. It examined their effects on cardiac function, systemic hemodynamics, renal perfusion, clinical decompensation, adverse events, and mortality. A further exploratory objective was to assess whether differences in treatment response could be interpreted according to cirrhosis severity and the cardiac or circulatory characteristics of the included populations. This analysis was intended to identify hypothesis-generating patterns and priorities for future phenotype-stratified trials, rather than to establish validated treatment-by-phenotype interactions.

## Review

Materials and methods

Study Design and Reporting Framework

This systematic review was designed to evaluate how pharmacological interventions that modify cardiac function, vascular tone, effective circulating volume, or natriuretic signaling influence cardiocirculatory and cardiorenal function in adults with cirrhosis. The review was conducted and reported in accordance with the Preferred Reporting Items for Systematic Reviews and Meta-Analyses 2020 (PRISMA 2020) statement [8]. The review process incorporated a predefined research question, structured eligibility criteria, systematic database searches, independent study selection, standardized data extraction, formal risk-of-bias assessment, and evidence synthesis. A PRISMA 2020 flow diagram was used to document the numbers of records identified, removed as duplicates, screened, assessed in full text, excluded with reasons, and included in the final synthesis. The review protocol was not prospectively registered in PROSPERO or another public repository. However, the research question, eligibility criteria, and planned methods were defined before formal study screening commenced.

Research Question and PICO Framework

The research question was structured according to the Population, Intervention, Comparator, and Outcomes (PICO) framework [9]. The population comprised adults with established cirrhosis of any etiology or severity, including compensated or decompensated cirrhosis, diuretic-responsive or refractory ascites, cirrhotic cardiomyopathy, impaired cardiac reserve, and left ventricular diastolic dysfunction. Eligible interventions included pharmacological strategies capable of modifying heart rate, beta-adrenergic activity, myocardial performance, vascular tone, effective circulating volume, or natriuresis. These interventions included beta-blockers, ivabradine, intravenous albumin, natriuretic peptides, and related cardiovascularly active therapies. Acceptable comparators included placebo, standard care, an active treatment, a clinically distinct cirrhosis phenotype, or pretreatment measurements from the same participants in prospective repeated-measures studies. Outcomes included cardiac reserve, systolic and diastolic function, cardiac output, heart rate, systemic and portal hemodynamics, renal perfusion, neurohormonal and cardiovascular biomarkers, renal dysfunction, hepatorenal syndrome-acute kidney injury (HRS-AKI), hepatic decompensation, hospitalization, adverse events, and mortality.

The intentionally broad scope reflected the review's focus on the shared cardiocirculatory consequences of cirrhosis rather than the comparative effectiveness of a single intervention. Although the eligible interventions acted through different mechanisms, each could alter the balance among cardiac performance, systemic vascular tone, effective arterial blood volume, and renal perfusion. This scope allowed treatment responses to be examined across clinically relevant hemodynamic phenotypes. Because the populations, interventions, and outcomes were not considered sufficiently comparable for estimation of a common treatment effect, no quantitative pooling or direct comparison between interventions was undertaken. Findings were instead synthesized according to therapeutic mechanism and underlying hemodynamic phenotype, and the conclusions were restricted to the specific populations represented by the included studies.

Information Sources and Search Strategy

MEDLINE via PubMed, Scopus, Web of Science Core Collection, and the Cochrane Central Register of Controlled Trials (CENTRAL) were searched for eligible studies published from August 1, 2016, through July 31, 2026. This 10-year period was chosen to focus the review on recent clinical evidence, as methods for assessing cardiac dysfunction in cirrhosis and the interventions under study have evolved. The restriction may have excluded older relevant studies and was considered when interpreting the findings. The final search was conducted on July 31, 2026. Controlled vocabulary, where supported by the database, and free-text terms addressed three principal concepts: cirrhosis, cardiocirculatory dysfunction, and relevant pharmacological interventions. Synonymous terms within each concept were combined with "OR", and the concept groups were combined with "AND". Table 1 and the Appendix present the database-specific search terms, fields, date limits, and Boolean structure. Reference lists of the included studies and relevant reviews were also screened for additional eligible studies.

**Table 1:** Search terms and Boolean approach used across the included databases. CENTRAL, Cochrane Central Register of Controlled Trials Database-specific controlled vocabulary, field specifications, and syntax were applied as appropriate.

Database	Search terms	Boolean approach
MEDLINE via PubMed	Liver cirrhosis, decompensated cirrhosis, refractory ascites; cirrhotic cardiomyopathy, cardiac dysfunction, cardiac reserve, diastolic dysfunction, hemodynamics, renal perfusion; beta-blockers, metoprolol, propranolol, carvedilol, ivabradine, albumin, ularitide, natriuretic therapy	Related terms were combined with OR; cirrhosis, cardiocirculatory dysfunction, and intervention groups were combined with AND
Scopus	Cirrhosis, decompensated cirrhosis, refractory ascites; cardiac dysfunction, cirrhotic cardiomyopathy, cardiac reserve, diastolic dysfunction, renal perfusion; beta-blockers, ivabradine, albumin, ularitide, natriuretic agents	OR within each group and AND between groups
Web of Science Core Collection	Cirrhosis, end-stage liver disease, refractory ascites; cardiomyopathy, cardiovascular dysfunction, cardiac output, hemodynamic dysfunction, renal perfusion; beta-blockers, heart-rate reduction, albumin, ularitide, natriuretic therapy	OR within each group and AND between groups
CENTRAL	Cirrhosis, decompensated cirrhosis, refractory ascites; cirrhotic cardiomyopathy, cardiac dysfunction, diastolic dysfunction, cardiac reserve; metoprolol, propranolol, carvedilol, ivabradine, albumin, ularitide	OR within each group and AND between groups

Eligibility Criteria

Eligible studies enrolled adults with established cirrhosis and evaluated a pharmacological intervention affecting cardiac function, systemic hemodynamics, vascular tone, effective circulating volume, or renal perfusion. Randomized and nonrandomized clinical trials, prospective interventional studies, pre-post studies, and eligible biomarker substudies were included. Studies were required to report at least one cardiac, hemodynamic, renal, or related clinical outcome. A formal diagnosis of cirrhotic cardiomyopathy was not mandatory because relevant trials also examined broader cardiocirculatory phenotypes in decompensated cirrhosis.

Retrospective or cross-sectional studies without an intervention, case reports, case series, reviews, editorials, insufficient conference abstracts, animal studies, and in vitro investigations were excluded. Studies involving noncirrhotic liver disease, hepatic dysfunction secondary to primary cardiac disease, portopulmonary hypertension, or hepatopulmonary syndrome were also excluded unless relevant cirrhosis-specific systemic hemodynamic data were separately available.

Study Selection

All retrieved records were imported into reference-management software, and duplicates were removed before screening. Two reviewers independently screened titles and abstracts against the predefined eligibility criteria. Articles considered potentially relevant by either reviewer underwent full-text assessment. The same reviewers independently evaluated the full texts and recorded the principal reason for excluding each ineligible study. Disagreements at either stage were resolved through discussion and consensus, with consultation from a third reviewer when agreement could not be reached. Multiple reports arising from the same clinical trial were linked and treated as a single study where they described overlapping populations, while distinct prespecified substudies were retained when they provided additional cardiovascular or biomarker outcomes.

Data Extraction and Outcomes

Data were extracted using a standardized form and independently verified by a second reviewer. Extracted information included author, publication year, study design, sample size, follow-up duration, cirrhosis stage, ascites phenotype, diagnostic cardiac criteria, intervention, dose, treatment duration, comparator, and cardiovascular assessment method. Cardiac and hemodynamic outcomes included stress-induced stroke-volume response, left ventricular diastolic dysfunction, E/e′, ejection intraventricular pressure difference, heart rate, cardiac output, hepatic venous pressure gradient, systemic blood pressure, renal perfusion pressure, and relevant neurohormonal or cardiovascular biomarkers. Renal and clinical outcomes included changes in estimated glomerular filtration rate, urine production, sodium excretion, AKI, hepatorenal syndrome-AKI (HRS-AKI), hepatic encephalopathy, hospitalization, adverse reactions, treatment discontinuation, and mortality. Numerical results were extracted as event counts, percentages, mean or median changes, correlation coefficients, odds ratios, hazard ratios, incidence-rate ratios, 95% confidence intervals, and P values, as reported by the original investigators.

Risk-of-Bias Assessment

Two reviewers independently assessed methodological quality using design-appropriate tools. Randomized trials and randomized trial substudies were evaluated using the Cochrane Risk of Bias 2 (RoB 2) tool [10] across the domains of randomization, deviations from intended interventions, missing outcome data, outcome measurement, and selection of the reported result. The prospective nonrandomized pre-post study was assessed using the Risk Of Bias In Non-randomized Studies of Interventions (ROBINS-I) tool, with particular attention to confounding, participant selection, intervention classification, deviations from intended interventions, missing data, outcome measurement, and selective reporting. The Quality In Prognosis Studies (QUIPS) tool was not formally applied because prognostic biomarker associations were not a primary review objective and were reported only as secondary exploratory findings within a randomized trial substudy [11]. These associations were not used to establish independent prognostic conclusions and were interpreted cautiously in view of participant selection, missing follow-up samples, multiplicity, and potential selective reporting. Disagreements were resolved through discussion and consensus. Risk-of-bias judgments were incorporated into the interpretation of the findings rather than used as an automatic basis for study exclusion.

Data Synthesis

A structured narrative synthesis was performed because substantial heterogeneity in populations, cardiac phenotypes, interventions, assessment methods, treatment duration, and reported outcomes precluded a clinically defensible meta-analysis. Studies were grouped according to the principal therapeutic mechanism, including beta-adrenergic blockade, targeted heart-rate reduction, modulation of effective circulating volume, and natriuretic or vasodilatory therapy. Findings were then interpreted according to the underlying hemodynamic phenotype, including impaired cardiac reserve, left ventricular diastolic dysfunction, sympathetic-dependent cardiac compensation, and mixed arterial underfilling with cardiac filling stress. Particular emphasis was placed on whether changes in cardiac or circulating markers were accompanied by concordant changes in renal perfusion, hepatic decompensation, adverse events, or survival. Statistical significance was not considered in isolation; effect direction, magnitude, precision, study design, risk of bias, and physiological plausibility were considered collectively when drawing conclusions.

Results

Study Selection Process

The systematic search identified 401 records, including 118 from MEDLINE through PubMed, 137 from Scopus, 103 from Web of Science Core Collection, and 43 from Cochrane CENTRAL. After removing 35 duplicates, 366 records underwent title and abstract screening, of which 212 were excluded. Full texts were sought for 154 reports, but 13 could not be retrieved, leaving 141 reports for eligibility assessment. Of these, 136 were excluded because they were noninterventional or cross-sectional studies (n = 36), ineligible publication types (n = 46), animal or in vitro studies (n = 22), noncirrhotic liver populations (n = 9), studies of hepatic dysfunction secondary to primary cardiac disease (n = 13), or studies focused on portopulmonary hypertension or hepatopulmonary syndrome (n = 10). Ultimately, five studies satisfied the eligibility criteria and were included in the qualitative synthesis, as presented in Figure 1.

**Figure 1:**
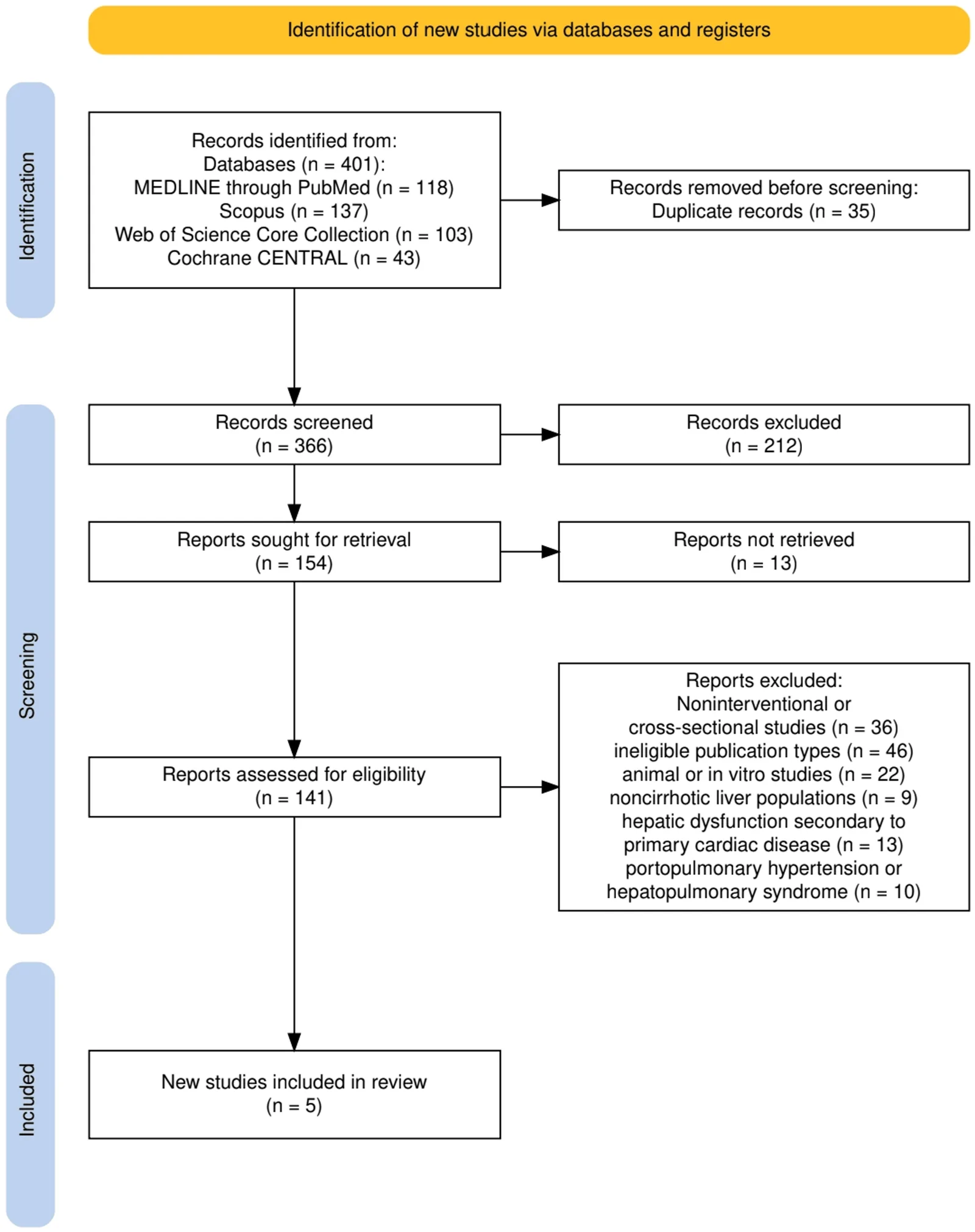
Flow diagram of study identification, screening, eligibility assessment, and inclusion. PRISMA: Preferred Reporting Items for Systematic Reviews and Meta-Analyses

Characteristics of the Selected Studies

Five studies published between 2018 and 2024 included 465 participants and comprised randomized trials, a prospective pre-post study, and a blinded biomarker substudy. Populations ranged from established cirrhotic cardiomyopathy and LV diastolic dysfunction to refractory ascites and hospitalized decompensated cirrhosis. Interventions included beta-blockers, ivabradine, ularitide, and targeted albumin, with follow-up ranging from 48 hours to 12 months. Outcomes covered cardiac function, systemic hemodynamics, renal perfusion, clinical decompensation, and mortality, as summarized in Table 2.

**Table 2:** Characteristics and principal findings of the included studies evaluating pharmacological cardiocirculatory interventions in cirrhosis. AKI, acute kidney injury; ANP, atrial natriuretic peptide; ATTIRE, Albumin To prevenT Infection in chronic liveR failurE; CCM, cirrhotic cardiomyopathy; CI, confidence interval; DRA, diuretic-responsive ascites; eGFR, estimated glomerular filtration rate; EIVPD, ejection intraventricular pressure difference; E/e′, ratio of early transmitral flow velocity to early diastolic mitral annular velocity; HR, hazard ratio; HRS-AKI, hepatorenal syndrome-acute kidney injury; HVPG, hepatic venous pressure gradient; IL-6, interleukin-6; IRR, incidence rate ratio; IV, intravenous; LV, left ventricular; LVDD, left ventricular diastolic dysfunction; MAP, mean arterial pressure; MELD, Model for End-Stage Liver Disease; NSBB, nonselective beta-blocker; NT-proBNP, N-terminal pro-B-type natriuretic peptide; OR, odds ratio; RA, refractory ascites; RPP, renal perfusion pressure; THR, targeted heart-rate reduction

Study	Design and follow-up	Population	Intervention and comparator	Cardiovascular phenotype/assessment	Cardiac and hemodynamic findings	Statistical data	Renal and clinical outcomes	Safety and interpretation
Silvestre et al., 2018 [12]	Prospective randomized placebo-controlled trial; six months	78 adults aged 18–60 years with cirrhosis and an abnormal cardiac-output response during dobutamine stress echocardiography; primary cardiac disease and alcohol history excluded	Metoprolol versus placebo	Cardiac reserve assessed by dobutamine stress echocardiography. Primary endpoint: >30% increase in left ventricular stroke volume during stress. Diastolic function, morphology, noradrenaline, renin activity, and troponin were also assessed	Metoprolol did not improve cardiac reserve, diastolic function, morphology, or biomarkers compared with placebo	Improved stress stroke-volume response: 3 (7.3%) versus 9 (24.3%), P = 0.057. Diastolic dysfunction changed from 2 (4.8%) to 5 (15.6%) with metoprolol and from 10 (27.0%) to 9 (31.0%) with placebo, P = 0.67. No significant differences in other echocardiographic or biomarker measures	Cirrhosis-related events, hospitalization, and mortality did not differ significantly; renal outcomes were not separately reported	Six months of metoprolol did not reverse impaired cardiac reserve or cardiac abnormalities in cirrhotic cardiomyopathy
Téllez et al., 2020 [13]	Prospective pre-post repeated-measures study; baseline and four-week assessments	38 patients with cirrhosis receiving nonselective beta-blocker prophylaxis: diuretic-responsive ascites, n = 18; refractory ascites, n = 20	Propranolol for four weeks; within-patient pre-post analysis with comparison between diuretic-responsive and refractory ascites	Ejection intraventricular pressure difference, heart rate, hepatic venous pressure gradient, cardiopulmonary pressures, renal perfusion pressure, estimated glomerular filtration rate, vasodilation, and sympathetic/inflammatory activation	Propranolol reduced heart rate and hepatic venous pressure gradient similarly in both groups but markedly suppressed compensatory systolic function in refractory ascites. This reduction was related to vasodilation and sympathetic activation	Baseline ejection intraventricular pressure difference: refractory ascites 4.5 (2.8–5.7) and diuretic-responsive ascites 4.2 (3.1–5.7) mmHg versus normal 2.4–3.6 mmHg. Change: −20% versus −2%, P < 0.01. In refractory ascites, the reduction correlated with vasodilation, nitric oxide, norepinephrine, and interleukin-6 (r > 0.40, all P < 0.05) and with reductions in renal perfusion pressure and estimated glomerular filtration rate (r > 0.40, all P < 0.01)	Renal function deteriorated only in refractory ascites. Renal perfusion pressure fell below the renal autoregulatory threshold in 11/20 patients (55%), including all four patients with hepatorenal syndrome-acute kidney injury	Nonselective beta-blockers may suppress an essential cardiac compensation in refractory ascites, lowering renal perfusion and impairing renal function. The nonrandomized design and absence of an untreated control limit causal certainty
Gantzel et al., 2024 [14]	Randomized placebo-controlled trial with 2:1 allocation; inpatient treatment for up to 48 hours; terminated early after interim analyses	17 patients with cirrhosis and refractory ascites: ularitide, n = 11; placebo, n = 6	Intravenous ularitide, initially 30 ng/kg/min and later reduced to 20 ng/kg/min, versus placebo	Systemic blood pressure and renal responsiveness; primary endpoint was urine production. Secondary endpoints included urinary sodium excretion and body weight; no cardiac imaging	Ularitide caused clinically important blood-pressure reductions that compromised renal responsiveness; no direct cardiac-function benefit was assessed	Mean urine production decreased from 47.5 to 22.8 mL/h, P = 0.04. Reported reduction with ularitide versus placebo: 24.7 versus −6.2 mL/h, P = 0.05. Adverse-reaction incidence-rate ratio: 8.5 (95% CI 2–35), P = 0.003	Ularitide did not increase urinary sodium excretion or prevent weight gain; hypotension was accompanied by reduced urine production	Ularitide provided no renal or ascites benefit and caused more adverse reactions. Findings demonstrate the hemodynamic cost of vasodilatory therapy in refractory ascites; interpretation is limited by the small sample and early termination
Premkumar et al., 2020 [15]	Randomized controlled trial; 12 months	189 of 260 screened patients with cirrhosis and left ventricular diastolic dysfunction: targeted heart-rate reduction group, n = 94; standard care, n = 95	Targeted heart-rate reduction using carvedilol with ivabradine added when required versus standard care without beta-blockers; target heart rate 55–65 beats/min	Left ventricular diastolic dysfunction grade, heart-rate response, E/e′, N-terminal pro-B-type natriuretic peptide, norepinephrine, plasma renin activity, and aldosterone	Targeted heart-rate reduction was achieved in most treated patients and was associated with improvement or reversal of left ventricular diastolic dysfunction. Neurohormonal and cardiac biomarkers decreased among responders; left ventricular diastolic dysfunction progressed in some controls	Targeted heart-rate reduction at four weeks: 88/94 (93%). Achieved with carvedilol alone in 48 (61.5%) and additional ivabradine in 40/46 (86.9%). Left ventricular diastolic dysfunction reversed in 16 (20.5%) and improved in 34 (35.4%); it progressed in 10 controls (10.5%). Biomarkers decreased in responders, all P < 0.01. Mortality predictors were Model for End-Stage Liver Disease score, HR 1.52 (95% CI 1.22–2.75), P = 0.034; E/e′, HR 1.28 (95% CI 1.23–2.42), P = 0.048; and nonresponse, HR 1.30 (95% CI 1.20–1.80), P = 0.046	Overall mortality: 21/189 (11.1%); reported as 6 (14%) versus 15 (18%), P = 0.240. No mortality occurred among 78 patients with persistent targeted heart-rate reduction, P < 0.001. Standard care was associated with greater odds of acute kidney injury, OR 4.2 (95% CI 2.8–10.5), P = 0.027, and encephalopathy, OR 6.6 (95% CI 1.9–9.7), P = 0.040	Targeted heart-rate reduction improved left ventricular diastolic dysfunction and was associated with fewer renal and hepatic complications. Randomized all-group mortality was nonsignificant; survival among persistent responders was a postrandomization analysis and requires cautious interpretation
China et al., 2022 [16]	Blinded biomarker substudy of the randomized ATTIRE trial; sampling at baseline, day 5, and day 10; mortality assessed through three months	143 hospitalized patients with decompensated cirrhosis selected from 777 ATTIRE participants: targeted albumin, n = 71; standard care, n = 72	Intravenous albumin targeted to serum albumin >30 g/L versus standard care; median albumin exposure approximately 200 versus 20 g	Renin, atrial natriuretic peptide, syndecan-1, heart rate, mean arterial pressure, and creatinine; inflammatory markers and albumin function also assessed; no echocardiography	Targeted albumin did not improve renin, atrial natriuretic peptide, syndecan-1, blood pressure, heart-rate variation, or creatinine. Higher baseline renin, atrial natriuretic peptide, and syndecan-1 were associated with three-month mortality	Day 1–5 changes with albumin versus standard care: renin −630.9 versus −18.32 pg/mL, between-group 95% CI −96.93 to 1,322; syndecan-1 −88.73 versus +487.3 pg/mL, 95% CI −358.4 to 1,511; atrial natriuretic peptide −78.04 versus −518.8 pg/mL, 95% CI −2,329 to 1,447; creatinine −4.9 versus −5.1 μmol/L, 95% CI −18.6 to 18.3. All confidence intervals crossed zero	Renal dysfunction: 12.7% versus 19.4%; infection: 29.6% versus 23.6%. Mortality with albumin versus standard care: 9.9% versus 13.9% at 28 days, 19.7% versus 23.6% at three months, and 31.0% versus 29.2% at six months; no significant treatment differences	Targeted albumin did not reverse cardiovascular or inflammatory abnormalities and did not improve renal or survival outcomes. Increased pulmonary edema was reported in the parent ATTIRE trial

Risk of Bias Assessment

Using the Cochrane Risk of Bias 2 (RoB 2) tool, the assessed randomized results from Silvestre et al. [12], Gantzel et al. [14], Premkumar et al. [15], and China et al. [16] were judged to raise some concerns. The nonrandomized study by Téllez et al. [13] had a moderate risk of bias under the ROBINS-I tool. The principal concerns included incompletely described allocation procedures in Silvestre et al. [12], inconsistent denominators in Premkumar et al. [15], and missing follow-up samples in the selected biomarker subset analyzed by China et al. [16]. Early termination after 17 participants limited the precision of Gantzel et al.'s findings [14]. The favorable survival finding from Premkumar et al.'s postrandomization responder analysis [15] was interpreted separately because of its vulnerability to selection and confounding bias. Objective cardiovascular and renal measurements reduced concerns about outcome measurement in several studies. Full domain-level judgments are presented in Table 3.

**Table 3:** Domain-level risk-of-bias assessment of the included studies. ROBINS-I, Risk Of Bias In Non-randomized Studies of Interventions; RoB 2, Cochrane Risk of Bias 2

Study and result assessed	Tool	Domain-level judgments	Overall judgment	Principal reason
Silvestre et al., 2018 [12] - cardiac reserve	RoB 2	Randomization: Some concerns; deviations from intended interventions: Low; missing outcome data: Some concerns; outcome measurement: Low; selection of reported result: Low	Some concerns	Allocation concealment was insufficiently described, and denominators varied across assessments. The stress-echocardiographic outcome was objectively measured.
Téllez et al., 2020 [13] - cardiac and renal hemodynamics after propranolol	ROBINS-I	Confounding: Moderate; participant selection: Moderate; intervention classification: Low; deviations from intended interventions: Low; missing data: Low; outcome measurement: Low; selection of reported result: Low	Moderate	The before-and-after design lacked an untreated comparison group, and baseline differences between ascites groups could affect the observed changes.
Gantzel et al., 2024 [14] - urine output, blood pressure, and adverse events	RoB 2	Randomization: Some concerns; deviations from intended interventions: Some concerns; missing outcome data: Low; outcome measurement: Low; selection of reported result: Some concerns	Some concerns	The ularitide dose was modified during the trial. Early termination after 17 participants also made the estimates imprecise.
Premkumar et al., 2020 [15] - randomized diastolic-function and clinical comparisons	RoB 2	Randomization: Some concerns; deviations from intended interventions: Some concerns; missing outcome data: Some concerns; outcome measurement: Low; selection of reported result: Some concerns	Some concerns	Inconsistent denominators and incomplete reporting limit confidence in the randomized comparisons. The persistent-responder survival analysis requires separate caution because responder status was determined after randomization.
China et al., 2022 [16] - cardiovascular biomarkers	RoB 2	Randomization: Low; deviations from intended interventions: Low; missing outcome data: Some concerns; outcome measurement: Low; selection of reported result: Some concerns	Some concerns	Biomarkers were assessed in a subset of ATTIRE participants; later samples were reduced by discharge or death, and multiple biomarker comparisons were reported.

Discussion

Principal Findings

The available clinical evidence indicates that therapeutic modulation of cardiocirculatory dysfunction in cirrhosis produces heterogeneous and context-dependent effects. Among patients with an impaired cardiac response to pharmacological stress, Silvestre et al. [12] found that six months of metoprolol did not restore cardiac reserve or improve cardiac morphology, neurohormonal activity, or cirrhosis-related outcomes. Conversely, Premkumar et al. [15] reported that targeted heart-rate reduction with carvedilol, supplemented by ivabradine when required, improved left ventricular diastolic dysfunction and was associated with fewer episodes of AKI and hepatic encephalopathy. However, mortality did not differ significantly between the randomized groups, and the favorable survival findings among persistent responders require cautious interpretation. In refractory ascites, Téllez et al. [13] demonstrated that propranolol suppressed sympathetic support of systolic function, reduced renal perfusion pressure, and impaired renal function. Gantzel et al. [14] similarly found that ularitide-induced reductions in blood pressure were accompanied by decreased urine production and more frequent adverse reactions. In the selected Albumin To prevenT Infection in chronic liveR failurE (ATTIRE) biomarker substudy of hospitalized patients with decompensated cirrhosis, China et al. [16] found that an albumin strategy targeting a serum concentration above 30 g/L did not meaningfully improve the measured cardiovascular biomarkers, renal outcomes, or survival. This finding should not be extrapolated to other albumin indications, dosing strategies, treatment durations, or cirrhosis populations. Collectively, these findings generate the hypothesis that treatment effects may vary according to cirrhosis severity, cardiac phenotype, and dependence on cardiovascular compensation. However, treatment-by-phenotype interactions have not been adequately tested in randomized clinical trials.

Cardiovascular Dysfunction as a Stage-Dependent Spectrum

The findings support a cautious interpretation of cardiocirculatory dysfunction in cirrhosis as a stage-dependent spectrum rather than a uniform state of myocardial impairment. The hyperdynamic circulation characteristic of advanced liver disease should not necessarily be interpreted as evidence of preserved cardiovascular health, since increased heart rate, cardiac output, and contractile activity may represent compensatory responses to systemic vasodilation and reduced effective arterial blood volume [17]. Early in this spectrum, enhanced cardiac activity may sustain organ perfusion despite substantial circulatory disturbance. With disease progression, subclinical diastolic dysfunction or impaired stress reserve may emerge, followed by increasing reliance on sympathetic activation to maintain cardiac output. When this compensation becomes inadequate, renal hypoperfusion and clinical decompensation may develop. The observations of Téllez et al. [13] are particularly informative in this context. Patients with refractory ascites demonstrated elevated baseline systolic function that was closely related to vasodilation and sympathetic activation, while propranolol-induced suppression of this response correlated with reductions in renal perfusion pressure and estimated glomerular filtration rate. Thus, apparently increased systolic activity in refractory ascites may be physiologically necessary rather than intrinsically beneficial. This framework may help explain why reducing cardiac activity improved ventricular filling in selected patients with diastolic dysfunction while compromising renal perfusion in patients with refractory ascites. However, this interpretation is based on comparisons across separate, heterogeneous studies rather than prospectively stratified randomized comparisons and should therefore be regarded as hypothesis-generating.

Reconciling the Divergent Beta-Blocker Findings

The apparently divergent beta-blocker findings may reflect differences in the cardiovascular function being targeted rather than a direct contradiction between studies. Silvestre et al. [12] evaluated patients with impaired cardiac reserve during dobutamine stress and found that metoprolol did not restore the stroke-volume response or improve cardiac morphology. Téllez et al. [13] studied patients with ascites and showed that propranolol suppressed a sympathetically mediated increase in systolic function that appeared important for maintaining renal perfusion, particularly in refractory ascites. In contrast, Premkumar et al. [15] examined patients with left ventricular diastolic dysfunction and reported improvement with a carvedilol-based targeted heart-rate strategy, supplemented by ivabradine when required. These studies differed in cardiac phenotype, cirrhosis severity, ascites status, beta-blocker selectivity, concomitant ivabradine use, treatment duration, and outcome definition. They also targeted distinct physiological processes, ranging from beta-adrenergic receptor blockade to controlled heart-rate reduction intended to improve diastolic filling. The mortality findings reported by Premkumar et al. [15] should nevertheless be interpreted cautiously because mortality did not differ significantly between the randomized groups, while the more favorable survival finding was derived from a postrandomization responder analysis. Overall, the effects of beta-blockade may depend on whether increased cardiac activity is maladaptive, neutral, or necessary to preserve perfusion at a particular stage of cirrhosis.

Why Volume Expansion and Natriuretic Therapy Did Not Correct the Circulation

The findings of China et al. [16] and Gantzel et al. [14] illustrate the limitations of treating decompensated cirrhosis as a simple disorder of insufficient circulating volume. In the ATTIRE biomarker substudy, China et al. [16] found that targeted albumin administration increased serum albumin exposure but did not meaningfully alter renin, atrial natriuretic peptide, syndecan-1, blood pressure, renal dysfunction, or mortality relative to standard care. Conversely, Gantzel et al. [14] found that ularitide, despite its anticipated natriuretic activity, produced clinically important reductions in blood pressure and urine output without improving sodium excretion or body weight. Although the ularitide trial was small and terminated early, its findings highlight the limited renal tolerance of additional vasodilation in refractory ascites. Together, these studies suggest that decompensated cirrhosis may involve the simultaneous presence of reduced effective arterial blood volume, systemic vasodilation, neurohormonal activation, increased cardiac filling stress, tissue congestion, and impaired renal autoregulation. The coexistence of elevated renin and atrial natriuretic peptide is particularly informative because it may reflect arterial underfilling alongside increased cardiac volume or filling pressure. In such a mixed physiological state, neither uniform plasma expansion nor additional natriuretic vasodilation is likely to correct every component of circulatory dysfunction [18].

The Cardiac-Renal Axis as the Functional Endpoint

The clinical significance of a cardiovascular intervention in cirrhosis depends not only on whether it modifies an echocardiographic or circulating marker but also on whether that change preserves renal perfusion and improves patient-level outcomes. Téllez et al. [13] demonstrated that reduced systolic compensation following propranolol treatment correlated with declines in renal perfusion pressure and estimated glomerular filtration rate, supporting a close relationship between cardiac adaptation and renal function in refractory ascites. In the trial by Gantzel et al. [14], treatment-associated hypotension was accompanied by reduced urine output, suggesting that the anticipated natriuretic effect could not be realized when systemic perfusion became inadequate. China et al. [16] similarly found that targeted albumin failed to improve either cardiovascular biomarkers or renal outcomes despite substantially greater albumin administration. Conversely, Premkumar et al. [15] reported fewer episodes of acute kidney injury among patients assigned to targeted heart-rate reduction, although this finding requires confirmation in independent trials with clearer adverse-event reporting. These observations support the integrated assessment of cardiac function, arterial pressure, systemic hemodynamics, renal perfusion, and clinical decompensation in future studies. Improvement in an isolated cardiac measurement should not be assumed to represent therapeutic benefit when renal function, perfusion pressure, or decompensation remains unchanged or worsens [19].

Toward Hemodynamic Phenotype-Guided Therapy

Based on the authors' synthesis of findings across the heterogeneous included studies, we propose a conceptual hemodynamic phenotype-guided framework. These phenotypes were not prospectively defined or formally tested in the included trials and should therefore be regarded as hypothesis-generating rather than established predictors of treatment response. An impaired cardiac-reserve phenotype may be characterized by an abnormal response to physiological or pharmacological stress despite relatively preserved resting function, raising the possibility that additional suppression of contractility could further limit cardiovascular reserve. A diastolic phenotype, characterized by left ventricular diastolic dysfunction, elevated E/e′, impaired filling, and excessive heart rate, might hypothetically respond differently to carefully controlled heart-rate reduction. A sympathetic-dependent phenotype may be present in refractory ascites with severe vasodilation and cardiac-output dependence, where beta-blockade could potentially reduce renal perfusion [19]. A mixed underfilling-congestion phenotype may be suggested by concurrent elevations in renin and atrial natriuretic peptide or other markers of cardiac filling stress, potentially creating vulnerability to both indiscriminate volume expansion and additional vasodilation. These proposed categories are not mutually exclusive, and patients may transition between or simultaneously express several phenotypes. No included randomized trial assigned treatment according to these phenotypes or formally tested treatment-by-phenotype interactions. Prospective validation is therefore required before this framework can inform clinical decisions [14,19].

Evidence Certainty and Review Limitations

The certainty of the available evidence is limited by several methodological and clinical considerations. Most studies enrolled relatively small populations, and the ularitide trial was terminated early, resulting in imprecise estimates and limited ability to evaluate uncommon clinical events. Participants differed substantially in cirrhosis severity, ascites phenotype, baseline cardiac function, and dependence on compensatory hemodynamic mechanisms. Cardiovascular assessment was similarly heterogeneous, ranging from stress echocardiography and intraventricular pressure gradients to conventional diastolic indices and circulating biomarkers, with limited use of contemporary strain imaging, cardiac magnetic resonance, or standardized definitions. The evidence base also included both randomized and nonrandomized designs, incomplete adverse-event reporting, postrandomization responder analyses, and biomarker analyses performed in selected trial participants. These differences precluded a reliable pooled quantitative synthesis and limited direct comparisons between interventions. Furthermore, not all studies required a formal diagnosis of cirrhotic cardiomyopathy. The broader term "cardiocirculatory dysfunction in cirrhosis" should therefore be used when interpreting the complete evidence base, while "cirrhotic cardiomyopathy" should be reserved for populations meeting an explicit diagnostic definition.

Future Research

Future studies should move beyond unselected treatment comparisons and prospectively evaluate whether baseline hemodynamic phenotype modifies therapeutic response. Multicenter trials should apply standardized definitions of cirrhotic cardiomyopathy and stratify participants according to cirrhosis severity, ascites status, resting cardiac function, stress reserve, diastolic indices, and markers of systemic vasodilation or congestion. Cardiovascular characterization should ideally integrate conventional echocardiography with global longitudinal strain, left atrial strain, stress testing, and cardiac magnetic resonance where feasible, while circulating renin, natriuretic peptides, troponin, and markers of endothelial injury may provide complementary biological information. Cardiac measurements should be evaluated alongside arterial pressure, systemic vascular resistance, renal perfusion, glomerular filtration, and clinically meaningful outcomes, such as HRS-AKI, hepatic decompensation, hospitalization, and mortality. Trials should also prespecify treatment-by-phenotype interactions, distinguish randomized comparisons from responder analyses, and report adverse events according to cirrhosis stage. Such studies could determine whether the proposed phenotypes represent reproducible treatment modifiers or merely different manifestations of advancing disease.

## Conclusions

Current evidence does not support a uniform cardiovascular treatment strategy for patients with cirrhosis. Cardiocirculatory dysfunction appears to encompass distinct and potentially overlapping phenotypes, including impaired cardiac reserve, diastolic dysfunction, sympathetic-dependent cardiac compensation, and combined arterial underfilling with cardiac filling stress. Interventions that improve one component of this circulation may provide no clinical benefit, or may become harmful, when they disrupt the mechanisms maintaining renal and systemic perfusion. Beta-blockade, targeted heart-rate reduction, albumin expansion, and natriuretic therapy should therefore be interpreted according to the physiological state in which they were studied rather than as universally beneficial or detrimental approaches. Although the available trials remain limited and do not yet justify phenotype-directed treatment recommendations, they provide an important conceptual direction for future research. The principal take-home message is that cardiovascular activation in cirrhosis is not always the abnormality requiring correction; in advanced disease, it may be the final compensation preserving organ perfusion. Effective therapy will depend on identifying when that response is maladaptive, when it is protective, and when intervention is more likely to restore hemodynamic balance than disturb it.

## Appendices

The searches combined three concepts: cirrhosis, cardiocirculatory dysfunction, and pharmacological intervention. Terms within each concept were combined with OR, and the three concepts were combined with AND. The publication period assessed was August 1, 2016, to July 31, 2026.

**Table 4:** Database-specific search terms and Boolean structure. Boolean structure: (cirrhosis terms joined by OR) AND (cardiocirculatory terms joined by OR) AND (intervention terms joined by OR). For databases using publication-year filters, records from the boundary years were assessed against the exact August 1, 2016, and July 31, 2026, dates during eligibility screening.

Database	Cirrhosis terms	Cardiocirculatory terms	Intervention terms and fields
MEDLINE via PubMed	"Liver Cirrhosis"[Mesh]; cirrho*[tiab]; "end-stage liver disease"[tiab]; "refractory ascites"[tiab]	"Cardiomyopathies"[Mesh]; "Hemodynamics"[Mesh]; "cirrhotic cardiomyopathy"[tiab]; "cardiac dysfunction"[tiab]; "cardiac reserve"[tiab]; "diastolic dysfunction"[tiab]; "cardiac output"[tiab]; hemodynamic*[tiab]; "renal perfusion"[tiab]	"Adrenergic beta-Antagonists"[Mesh]; "Albumins"[Mesh]; "Natriuretic Peptides"[Mesh]; metoprolol[tiab]; propranolol[tiab]; carvedilol[tiab]; ivabradine[tiab]; albumin[tiab]; ularitide[tiab]; "beta-blocker*"[tiab]; "natriuretic peptide*"[tiab]. Publication-date range: 2016/08/01:2026/07/31[dp].
Scopus	cirrho*; "end-stage liver disease"; "refractory ascites"	"cirrhotic cardiomyopathy"; "cardiac dysfunction"; "cardiovascular dysfunction"; "cardiac reserve"; "diastolic dysfunction"; "cardiac output"; hemodynamic*; "renal perfusion"	"beta-blocker*"; metoprolol; propranolol; carvedilol; ivabradine; albumin; ularitide; "natriuretic peptide*"; "heart-rate reduction". Terms searched in title, abstract, and keywords using TITLE-ABS-KEY.
Web of Science Core Collection	cirrho*; "end-stage liver disease"; "refractory ascites"	"cirrhotic cardiomyopathy"; "cardiac dysfunction"; "cardiovascular dysfunction"; "cardiac reserve"; "diastolic dysfunction"; "cardiac output"; hemodynamic*; "renal perfusion"	"beta-blocker*"; metoprolol; propranolol; carvedilol; ivabradine; albumin; ularitide; "natriuretic peptide*"; "heart-rate reduction". Terms searched in the Topic field (TS); publication years 2016–2026 (PY).
CENTRAL	MeSH descriptor [Liver Cirrhosis]; cirrho*; "end-stage liver disease"; "refractory ascites"	MeSH descriptor [Cardiomyopathies]; "cirrhotic cardiomyopathy"; "cardiac dysfunction"; "cardiac reserve"; "diastolic dysfunction"; "cardiac output"; hemodynamic*; "renal perfusion"	MeSH descriptors [Adrenergic beta-Antagonists], [Albumins], and [Natriuretic Peptides]; metoprolol; propranolol; carvedilol; ivabradine; albumin; ularitide; "beta-blocker*"; "natriuretic peptide*". Free-text terms searched in title, abstract, and keywords (:ti,ab,kw).
